# Survival time and predictors of death among HIV infected under five children after initiation of anti -retroviral therapy in West Amhara Referral Hospitals, Northwest Ethiopia

**DOI:** 10.1186/s12887-022-03693-5

**Published:** 2022-11-21

**Authors:** Gebrie Getu Alemu, Zelalem Mehari Nigussie, Baye Tsegaye Amlak, Anemaw Asrat Achamyeleh

**Affiliations:** 1 Department of Nursing, Bahir Dar Health Science College, Bahir Dar, Ethiopia; 2 Department of Epidemiology and Biostatistics, School of Public Health, College of Medicine and Health Sciences, Bahir Dar University, Bahir Dar, Ethiopia; 3 College of Health Sciences, Department of Nursing, Debre Markos University, Debre Markos, Ethiopia

**Keywords:** Anti-retroviral therapy, Under-five children, Survival status, Predictors, Ethiopia

## Abstract

**Background:**

Acquired immune deficiency syndrome is an infectious disease caused by the human immunodeficiency virus (HIV) that primarily targets an individual's immune system. In Ethiopia, nearly 24% of HIV-related deaths occur in children under the age of five. However, studies regarding the survival time of HIV-positive under-five children after anti-retroviral therapy initiation are limited with poor evidence of predictors of death.

**Objective:**

To assess survival time and predictors of death among HIV infected under-five children after initiation of anti-retroviral therapy in West Amhara Referral Hospitals, Northwest Ethiopia, 2021.

**Methods:**

A multicenter institution-based retrospective follow-up study was conducted among 432 HIV-positive under-five children on anti-retroviral therapy selected by simple random sampling from January 2010 to December 2019. A standardized data extraction tool was employed, which was adapted from anti-retroviral therapy entry and follow-up forms. The event of interest for this study is death, whereas the absence of experience of death is censored. Data were entered into Epi-Data version 3.1 and exported to STATA version 14. The Kaplan–Meier curve was used to estimate the survival probability. The Cox regression model was used to identify independent predictors of death.

**Results:**

Among the 415 records included in the final analysis, 25 (6.02%) of the individuals were died. The incidence rate of death was found to be 2.87 per 1000 child-months (95%CI: 1.94–4.25). The cumulative survival probabilities of children after 6, 12, 24, and 36 months were 0.97, 0.95, 0.92, and 0.85 respectively. HIV-infected under-five children who lived in rural areas (AHR 3.32:-95% CI 1.17–9.39), with poor adherence to anti-retroviral therapy (AHR = 3.36; CI: 1.06, 10.69), without Isoniazide prophylaxis (AHR = 3.15; CI: 1.11, 8.94) and with anemia (AHR: 3.05, 95% CI: 1.16, 8.03) were at higher risk of death.

**Conclusion and recommendation:**

Death of HIV-infected under-five children on anti-retroviral therapy is high within the first one year after enrolment. Living in rural area, had poor adherence, lacked Isoniazide prophylaxis, and anemia were predictors of death. Therefore, clinicians shall emphasize for those specific risk factors of death and take action accordingly.

## Introduction

Acquired Immune Deficiency Syndrome (AIDS) is an infectious disease caused by Human Immune Virus (HIV), which primarily targets an individual's immune system [1]. It remains one of the leading causes of death among children under the age of five [2]. HIV/AIDS is a life-threatening communicable disease with a poor prognosis [3]. Antiretroviral Therapy (ART) is a medication treatment that helps patients infected with HIV to survive longer and healthier. The survival time is the length of time people were followed from the moment they started ART until they died, lost to follow up, or alive at the end of the follow up time [4].

Alarmingly, two out of five children living with HIV worldwide are unaware of their status. HIV infection rates among children have been dramatically rising in recent years [5]. The majority of under-five children living with HIV are infected via mother-to-child transmission (MTCT) during pregnancy, childbirth, or breastfeeding. It is believed that more than 90% of all HIV infections in children are transmitted through MTCT [6].

Children and adults have different HIV natural histories and clinical presentations, with children having a faster disease progression in infancy and a preference for recurring, common viral and bacterial infections over opportunistic illnesses. Undeveloped immune systems as well as the effects of viral replication and inflammation on somatic and neuro-developmental growth are blamed for the faster progression. The opportunistic infections (OIs) that emerge from immunological weakness in HIV-positive children under the age of five are frequently primary infections rather than reactivation diseases, resulting in higher mortality [1, 7, 8]. In Ethiopia, the first case of HIV was reported in 1984. Since then, HIV/AIDS has become a major public health concern in the country, resulting in millions of deaths [9].

Untreated infants infected with HIV in their first year of life have a significant mortality rate, making early HIV testing, timely results, and treatment beginning critical. All under-five children who visit a health facility should be offered HIV testing and counseling. Infants born to HIV-positive mothers who are at high risk of contracting the virus should be given dual prophylaxis with Zidovudine (AZT) twice daily and Nevirapine (NVP) once daily throughout the first six weeks of life regardless of whether they are breastfed or formula fed [8].

Globally, HIV is still a major public health concern, although it is particularly prevalent in low and middle-income countries (LMICs). AIDS continues to be the greatest cause of death among children [6]. Alarmingly, over half of children with HIV are receiving antiretroviral therapy, and two out of every five children living with HIV around the world are uninformed of their status. According to a research released today by UNICEF, at least 300,000 children, or one every two minutes, will become HIV positive for the first time in 2020. During the same time span, 120,000 additional children died from AIDS-related causes, or one child every five minutes [5]. Studies in Africa have shown that children taking antiretroviral therapy have a short survival time [4].

Perinatally infected children have high rates of treatment failure and drug resistance, which can make long-term treatment more difficult and lead to early mortality [10]. HIV exposure at birth has a greater impact on child mortality, especially in the first year of life and reduces children’s survival [11].

Despite significant improvements to antiretroviral treatment accessibility, the death of children on ART remains a persistent problem in Sub-Saharan African (SSA) countries [12]. In HIV-infected children in developing nations, the rate of disease progression and death is high [13]. Children under the age of five continue to be a major health issue in SSA. HIV/AIDS is one of the main reasons for the region's relatively slow drop in under-five mortality [14].

ART is well known for reducing AIDS-related mortality, while mortality has become a public health concern in Ethiopia [15]. Even though visible efforts to improve HIV-infected child survival have resulted in significant reductions in mortality rates among under-five children, the presence of persistent and intolerably high numbers of child deaths indicates that more work needs to be done to address the specific survival needs of children in Ethiopia [16].

HIV/AIDS has taken the lives of millions of people and left hundreds of thousands of children orphaned. Ethiopia's government has taken a number of initiatives to prevent the disease from spreading further, as well as to improve HIV care, treatment, and support for those living with HIV [8]. The country has met the majority of the Millennium Development Goals (MDGs) relating to HIV/AIDS. The rate of HIV/AIDS deaths, on the other hand, has been slow to diminish. Between 1990 and 2016, the annualized HIV/AIDS mortality rate reduction was only 0.4% [17]. Therefore, this study will be important to guide evidence-based information for better treatment and prevention of HIV/AIDS in resource-limited settings such as Northwest Ethiopia by assessing the survival time and predictors of death that have a role in the mortalities of HIV positive under-five children after the initiation of ART.

## Methods and materials

### Study design

A multicenter institution-based retrospective follow-up study was conducted in West Amhara Referral Hospitals, Northwest Ethiopia.

### Study area and study period

An institution-based retrospective follow-up study was conducted on under-five children who initiated ART in the period from 2010 to 2019 in the West Amhara Referral Hospitals ART center clinic, with the data abstraction (collection) time of September to October 2021. The West Amhara Referral Hospitals that gave ART in the follow up period were: University of Gondar Comprehensive Specialized Hospital (UoGCSH), Felege Hiwot Comprehensive Specialized Referral Hospital (FHCSRH), Debre Tabor Referral Hospital (DTRH), and Debre Markos Referral Hospital (DMRH). The University of Gondar Comprehensive Specialized Hospital is located in the North Gondar administrative zone, Amhara National Regional State, Ethiopia, which is about 738 km Northwest of Addis Ababa (the capital city of Ethiopia). Since 2005, when the hospital started ART, about 285 under-five children were enrolled, of which 198 HIV-infected under-five children were receiving active ART follow-up between January 2010 and December 2019.

The Debre Tabor Referral Hospital is the largest hospital in South Gondar, which is located 660 km away from Addis Ababa, the capital city of Ethiopia. To date, a total of 120 HIV-infected under-five children commenced ART at this site, of which 92 HIV-infected under-five children are receiving active ART during the follow-up period.

Felege Hiwot Referral Hospital is found in Bahir Dar, the capital of Amhara Regional State, which provides service for the Amhara Region and serves over 12 million people from the surrounding area. To date, a total of 297 HIV-infected under-five children commenced ART at this site, of which, about 210 HIV-infected under-five children are receiving active ART follow-up between January 2010 and December 2019.

Debre Markos town is located 300 km far from Addis Ababa, the capital city of Ethiopia, and 265 km far from Bahir Dar, the capital city of the Amhara Region. To date, a total of 225 HIV-infected under-five children commenced ART at this site, of which about 148 HIV-infected under-five children are receiving active ART follow-up between January 2010 and December 2019.

### Source and study population

All under-five children with HIV infection ever enrolled in pediatrics ART clinic and initiated ART in West Amhara Referral Hospitals providing ART services.

### Inclusion criteria

All under-five HIV-positive children who were enrolled in the Pediatric ART Clinic from 2010 to 2019 in the study hospitals who took ART medication for at least three months were included.

### Exclusion criteria

Under-five children with incomplete records of age and ART initiation date during the data collection period were excluded.

### Sample size determination

Sample size was determined by using STATA version 14 statistical software by considering the proportionate allocation of events in a log rank test. Significant predictors of mortality in HIV-infected under-five children on ART were identified in the literature, and software calculations were performed using 95% confidence intervals, 85% power, 5% level of significance, and a 10% withdrawal assumption, yielding a total sample size of 432 for this study.

### Sampling technique and procedure

Simple random sampling was used to select the samples from each hospital. Based on their respective source populations, a proportionally allocated sample was calculated for the selected hospitals. A sampling frame was prepared for all referral hospitals independently from the patients’ medical registration numbers. Patients’ medical registration numbers were collected from the Health Management Information System (HMIS) ART registration book and then entered into Microsoft Excel and then copied and pasted into SPSS version 23.

**Fig. 1:**
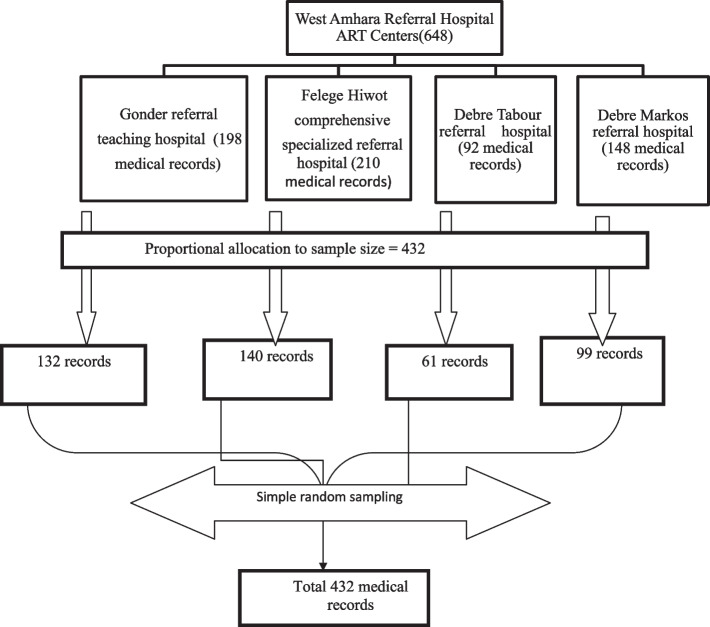
Schematic presentation of the sampling procedure to assess the survival time and predictors of death among HIV-infected under-five children on ART in the West Amhara Referral Hospitals ART center, Northwest Ethiopia, from January, 2010 to December, 2019

SPSS was used to generate random numbers for patients’ medical registration numbers. The required folders were selected among the medical registration numbers for each institution independently. Then the calculated sample size was allocated to the four referral hospitals (Fig. 1).

### Data collection tools and procedure

A standardized data extraction checklist was carefully developed after reviewing some HIV-infected children's charts. Data were extracted from the document by using structured checklists that were adapted and prepared based on the charts. Those under-five children with HIV who initiated ART from January 2010 to December 2019 and had follow-up within the selected hospitals were retrieved. The data extraction tool was prepared for the collection of socio-demographic, clinical, laboratory, treatment, and outcome-related information that was important for the assessment. Data were collected by four BSc nurses under the supervision of two senior BSc nurses. Initially, the medical charts of eligible HIV-positive under-five children on ART were gathered from the card room using the patient’s medical registration number. Then, basic socio-demographic, clinical, and treatment-related variables of HIV-infected under-five children were extracted from those selected charts. The most recent laboratory test results and clinical findings prior to ART initiation were considered as a baseline value. However, variables like a history of treatment failure, history of regimen change, level of adherence, and history of OIs were taken on a follow-up basis. Besides, relevant patient data that was not available on the patient's chart was retrieved from the ART smart care file. Lastly, the consistency between records and collected data was confirmed by the principal investigator through randomly selected reviews of previously extracted medical records.

### Variables

#### Dependent variable

The child's death at any time.

#### Independent variables

The independent variables that were used to affect the time to death of under five children on ART are the following:

##### Sociodemographic Factors

These are children: -Age and sex.

##### Care giver

Age, sex, residence, relationship of care giver to the child, and status of parents.

##### Baseline clinical, laboratory, and immunological factors

These are predictors such as WHO clinical stage, TB and its treatment, hemoglobin level, CD4 count/percentage, ART regimen, nutritional status (underweight, wasting, stunting), OIs, and eligibility criteria for HAART.

Cotrimoxalzole preventive therapy (IPT), and Isoniazed Preventive Therapy (IPT), ART regimen change, treatment failure, child adherence to ART, and history of OIs are independent variables of the study.

### Operational definitions

#### Advanced WHO clinical stages

When HIV-infected children under the age of five are enrolled in ART, their clinical stages are stage III and IV [18].

#### Anemia

The hemoglobin level is less than 10 mg/dl [4].

#### Event

The event of interest for this study is the death of HIV-infected under-five children after the initiation of ART due to HIV, its complications, and OIs.

#### Censored

When a child is lost-to-follow-up, transferred out, or dies for reasons unrelated to the study (dies due to another cause) and lives longer than the follow-up period (not died at the end of their fifth year).

#### Time to event

The time interval (months) between ART initiations and the occurrence of death.

#### Survival

Absence of experience of death.

#### Follow-up period (time)

The time from the beginning of the study (January 1, 2010) to an event (death), loss to follow up, withdrawal from the study, not dying at the end of their fifth year until December 31/2019, and who additionally followed through data collection time for about 20 months of follow up time (for an average of 20 months of follow up time).

#### Incidence of death

The rate of child death during the follow-up time to the total person-months of observation.

#### Mild WHO clinical stages

Are stage I and II baseline clinical stages of HIV-infected under-five children during ART enrolment [18].

#### Under nutrition

Was defined as the child's having either a height for age (H/A) Z-score 2, or weight for age (W/A) Z-score 2, or weight for height (W/H) Z-score 2 standard deviations (SD) [19].

**Moderate underweight** was defined as children having a W/Age Z-score <  − 2 SD [19].

**Severe underweight** was defined as children having a W/Age Z-score <  − 3 SD [19].

**Moderate stunting** was defined as children having an H/Age Z-score <  − 2 SD [19].

**Severe stunting** was defined as children having H/Age Z-score <  − 3 SD [19].

**Moderate wasting** was defined as children having a W/H Z-score <  − 2 SD [19].

**Severe wasting** was defined as children having a W/H Z-score <  − 3 SD [19].

#### Opportunistic infections

When HIV infected child develops any form of OI after ART initiation during the follow-up period [20].

#### Treatment failure

**Clinical Failure:** After 6 months of effective therapy, a new or repeated clinical event shows advanced or severe immunodeficiency (WHO clinical stage 3 and 4 clinical conditions with the exception of tuberculosis). **Immunologic failure:** A persistent CD4 count of less than 200 cells/mm3 or < 10% (Persistent is to mean at least 2 CD4 measurements below the threshold). **Virologic failure:** occurs when two consecutive viral load measures with adherence support between measurements are greater than 1000 copies/ml within a 3-month interval [8].

#### Loss to follow-up

Children who have missed their follow-up or drug pick-up appointments for three months and more [21].

#### Transferred out

Those children who were transported to different medical facilities [21].

**An incomplete card is** considered when the indicator of the dependent variable and/or 5% of the independent variable is not registered.

The developmental status of a child is classified as **appropriate** (ability to achieve milestones for age), **delayed** (inability to achieve milestones for age), or **regression** (loss of what has been attained for age) [22].

#### CD4 counts or percentage (%)

Below the threshold was considered if the child had CD4 cell counts < 1500/ mm3 or 25% for age < 12 months, CD4 cell counts < 750/ mm3 or < 20% for age 12–35 months, CD4 cell counts < 350/mm3 or < 15% for age 36–59 months [23].

#### Adherence

**Good (> 95%)—**if the percentage of missed doses is ≤ 2 doses of 30 doses or ≤ 3 doses of 60 doses**; Fair: (85–94%)** if the percentage of missing doses is between 3 and 5 of 30 doses or 4–9 of 60 doses**; poor:** (< 85%**)** if missed doses are 6 doses of 30 doses or 10 and above doses of 60 doses, as documented by the ART physician [24].

### Data quality assurance and control

Training and orientation for the supervisors and data collectors were given for one day about sample size, data extraction, ethical issues, and methods of supervision. Before entering data, the collected data were reviewed and checked for completeness, and any incomplete data were discarded. Epi Data was used for data entry to prevent data entry errors.

### Processing and analysis of data

A descriptive analysis was carried out to present the magnitude of each study variable. The Kaplan–Meier survival curve was used to estimate survival time after ART initiation, and log rank tests were used to compare the survival curves. The probability of survival within each time interval and the cumulative probability of survival for each subsequent time interval were estimated using the life table.

The goodness of fit for the Cox regression model was checked by the Cox-Snell residual plot. Graphical tests such as (Log–log survival probability plot, Schoenfeld residual plot) and Schoenfeld residual global proportional hazard test were used to assess the proportional hazard assumption of the model. The parallelism of lines in the Log log survival probability plot, the presence of almost a constant smoother line on the Schoenfeld residual plot, and a *P*-value of > 0.05 on the Schoenfeld residuals Proportional hazard (PH) test were used to ascertain whether the proportional hazard assumption is satisfied or violated. A bivariable (simple) Cox proportional hazard model was used to identify candidate variables for multivariable Cox regression. A multivariable (multiple) Cox proportional hazard model was used to identify factors associated with time to death of HIV-infected under-five children. Variables having a *p*-value of less than 0.25 in the bivariable analysis were considered in the multivariable Cox regression analysis. Finally, the adjusted hazard ratio (AHR) with 95% CI was computed, and variables with a *p*-value less than 0.05 in the multivariable Cox regression analysis were considered as significant predictors of death. In addition, the outcome status was calculated by dividing the total number of occurrences during the follow-up period by the total number of observations. The incidence density was measured with a person's months of observation.

### Ethical considerations

Ethical approval for this study was obtained from the Bahir Dar University College of Medicine and Health Science Ethical Review Board. Permission letters were obtained from each hospital administration of the four participating entities. Since the study was a review of medical records, individual patients were minimally at risk for harm as confidentiality was likely to be achievable. To maintain confidentiality, collected data were coded and locked in a separate room. In addition, the data collection forms did not include names or unique ART numbers. This study was conducted in accordance with the Declaration of Helsinki.

## Result

### Socio-demographic characteristics

After reviewing 432 HIV-infected children's records, 415 records were included in the final analysis as 17 records were excluded due to incompleteness. About half, 210 (50.6%) of the study participants were females. The majority of participants, 285 (68.67%), were from urban areas, and 350 (84.34%) of the children were living with their parents. The baseline median age of the participant was 34 months (IQR: 21–48 months), with the median age of the caregiver was reported as 31 years (IQR: 27–37 years), and the majority of parents, 327 (77.35%), were both alive.

### Baseline and follow-up clinical, immunological, laboratory, and treatment-related characteristics

Of the total 415 under five-children, nearly two third, 261 (62.89%) of the study participants had baseline OIs. In the study participants regarding to the developmental milestone, 360 (86.75%), 47 (11.32%), and 8 (1.93%) were appropriate, delayed, and regressed, respectively.

Regarding the WHO clinical staging, less than half, (46.27%) of the study participants had advanced baseline WHO clinical Stage (3 and 4). About half, 212 (50.85%) of the respondents were screened for tuberculosis (TB) in the past (before the start of ART). Among them, 26 (6.27%) were positive for TB screening, and all of them were given TB treatment.

In this study, The key requirements for starting HAART among participants were both their baseline CD4 + cell count and WHO clinical stages, 178 (42.89%), followed with test and treat approach, 113 (27.23%). Moreover, more than half, 231 (55.66%) of the children had CD4 counts below the threshold. Furthermore, 51 (12.29%) of the participants had anemia at ART initiation. Eighty-three (20%) of the participants on ART had a history of treatment failure.

During the study follow-up period, 319 (76.87%) and 57 (13.73%) of the participants had good and fair adherence, respectively. Out of 39 (9.40%) study participants who had poor adherence, 8 (32%) died by the end of the follow-up period. Throughout the follow-up time, 178 (42.89%) of the study participants developed OIs. Regarding prophylaxis use, 372 (89.64%) of the participants were ever on Co-trimoxazole Preventive Therapy (CPT), whereas 198 (47.71%) were ever on Isoniazid Preventive Therapy (IPT). Regarding baseline nutritional status, 18.07%, 17.83%, and 18.07% of HIV-infected under-five children were severely underweight, stunted, and wasted, respectively.

**Fig. 2:**
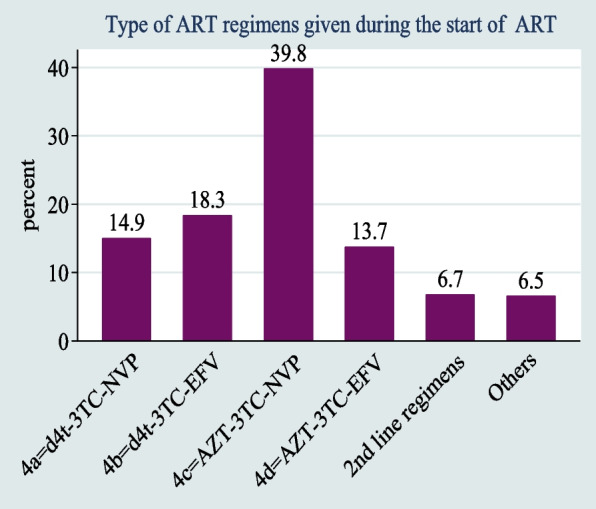
ART regimen for children under the age of five who began ART in West Amhara Referral Hospitals between January 2010 and December 2019

The commonest regimens that were given at the start of ART in the cohort are presented in Fig. 2. The regimen 4c = AZT-3TC-NVP was given to 165 (39.76%) children, and the regimen 4b = d4t-3TC-EFV was given to 76 (18.31%) children. ABC + 3TC + LPV/r, AZT + 3TC + LPV/r, AZT + 3TC + DRV/r^g^, ABC + 3TC + DTG, and AZT + 3TC + DTG were also taken as second line regimens (Fig. 2).

**Fig. 3:**
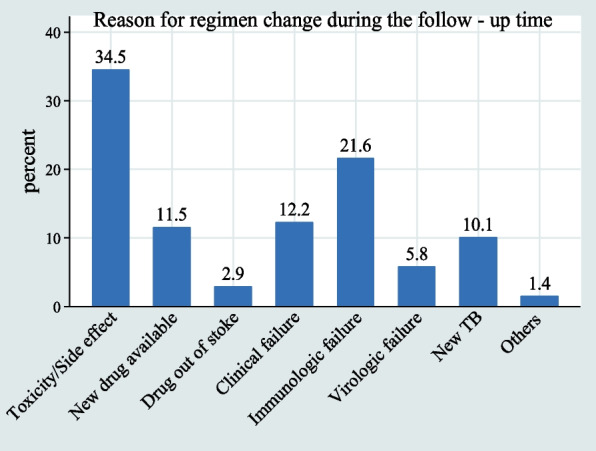
Reasons for ART regimen changes in children under the age of five who began ART in West Amhara Referral Hospitals between January 2010 and December 2019

During the follow-up period, one-third (33.5%) of HIV-infected children under the age of five had a history of ART regimen change. Of these, 48 (34.5%) were due to drug toxicity or side effects, followed by immunological failure of 30 (21.6%) (Fig. 3).

**Fig. 4:**
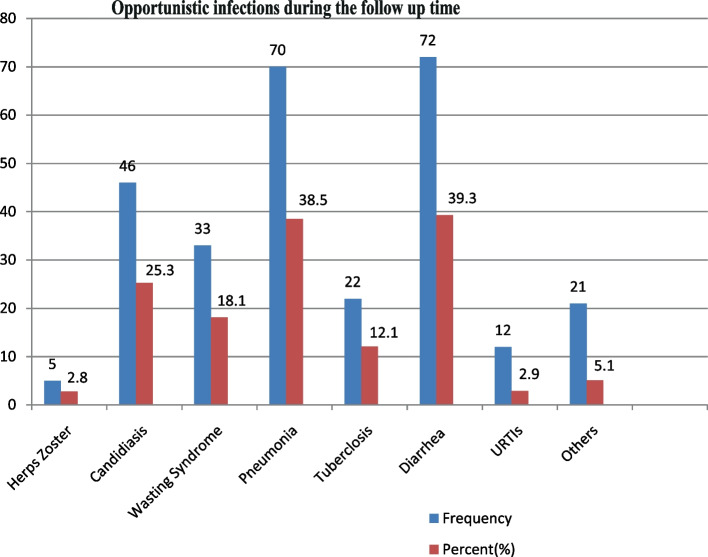
Opportunistic infections during the follow-up time of HIV-infected under-five children receiving ART in West Amhara Referral Hospitals from January 2010 to December 2019

About 42.89% of HIV-positive under-five children had OIs during the follow-up time. Of these, 39.3, 38.5, and 25.3% of the children had diarrhea, pneumonia, and candidiasis, respectively (Fig. 4).

### Survival characteristics after initiation of ART

**Fig. 5:**
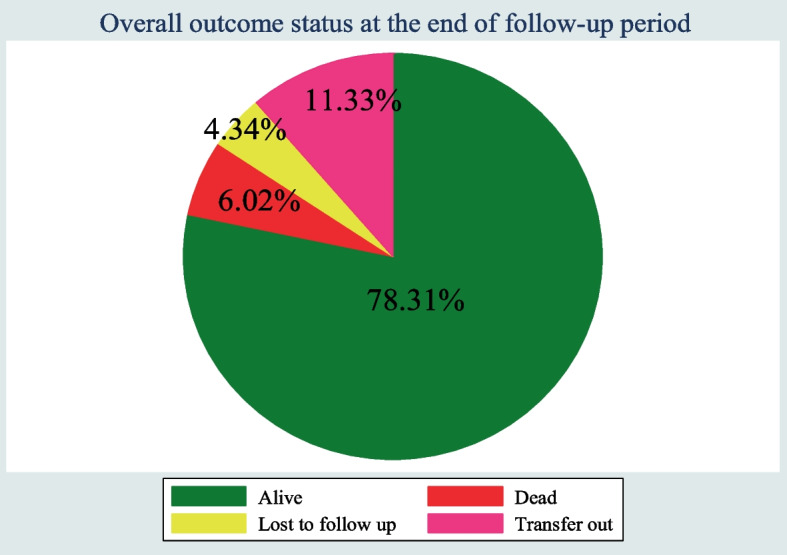
Outcome Status for HIV infected under five children receiving ART in West Amhara Referral Hospitals between January 2010 and December 2019

After the initiation of ART, HIV-infected children were followed for 3 months to 48 months, which provides a total of 8700.5 person-months (725.04 person-year) of observation. At the end of the follow up period, 325 (78.31%) of the children were alive; 18 (4.34%) were lost to follow up; 47 (11.33%) were transferred out to other health facilities; and 25 (6.02%) were reported dead due to HIV/AIDS (Fig. 5). Of the 25 deaths, 3 (12%) occurred in the first six months of follow-up and 10 (40%) occurred within the first 12 months of ART initiation. The incidence rate of death was 2.87 deaths per 1000 child-months (95%CI: 1.94–4.25) during the follow-up period.

The median follow-up period for children under the age of five after starting ART was found to be 19 months (IQR = 11–32). The median time to death was 14 months (IQR = 10–23). The cumulative probability of survival of under-five children on ART after the last month of follow-up was 85% (95%CI; 76.42–91.25). The cumulative survival probabilities of children after 6, 12, 24, and 36 months were 0.97, 0.95, 0.92, and 0.85, respectively.

**Fig. 6:**
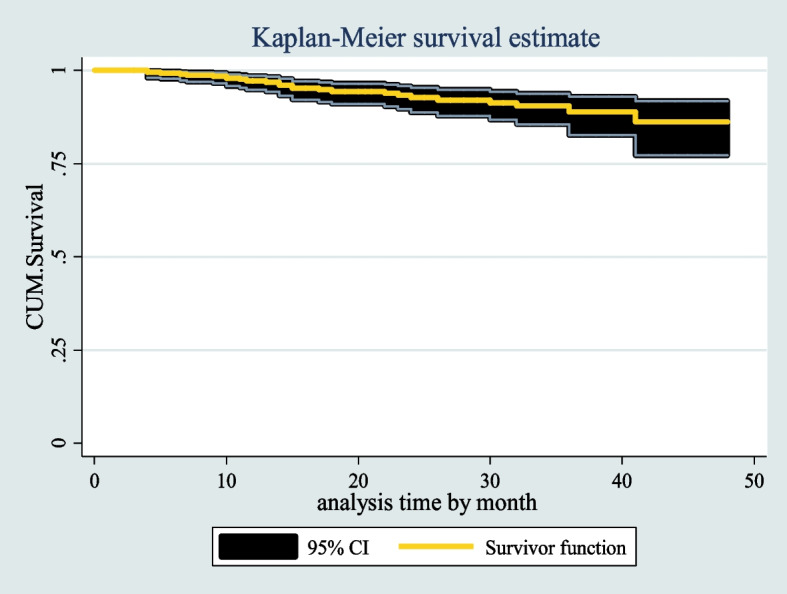
the overall Kaplan Meier survival curve with 95% confidence intervals of HIV infected under five children receiving ART in West Amhara Referral Hospitals between January 2010 and December 2019

The overall Kaplan–Meier survivor function estimate shows that most deaths have occurred in the early months of ART initiation. Based on the findings, of the total of 25 deaths, 3 (12%) of them occurred in the first six months of follow-up and around 10 (40%) deaths occurred in the first 12 months of follow-up, which then declined steadily through follow-up time and continued steadily in the later months of follow-up (Fig. 6).

**Fig. 7:**
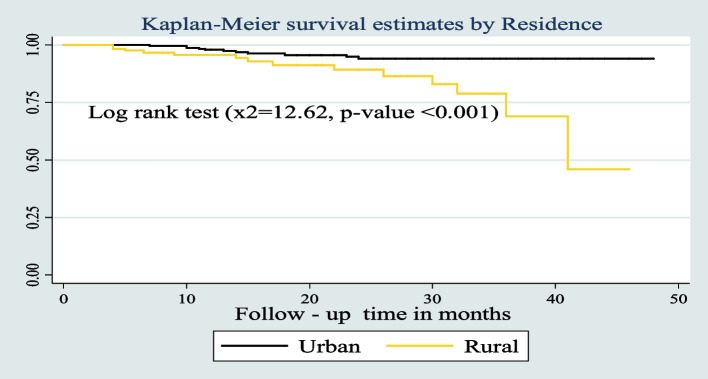
Kaplan–Meier survival estimates based on their residence during the follow up time among under five children on ART in West Amhara Referral Hospitals between January 2010 and December 2019

**Fig. 8:**
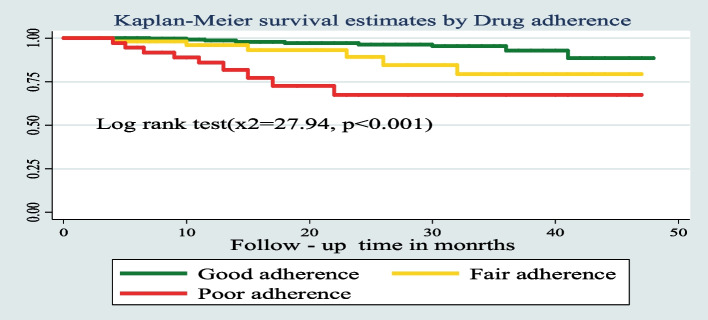
Kaplan–Meier survival estimates based on their drug adherence during the follow up time among under-five children on ART in West Amhara Referral Hospitals between January 2010 and December 2019

**Fig. 9:**
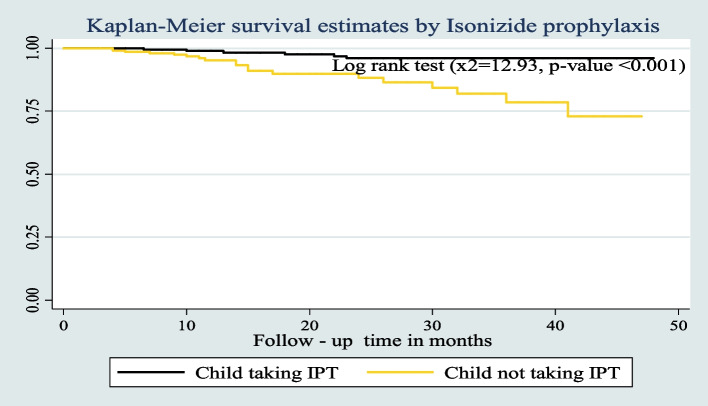
Kaplan–Meier survival estimates based on their Isoniazide Preventive Therapy (IPT) during the follow up time among under- five children on ART in West Amhara Referral Hospitals between January 2010 and December 2019

The survival probability of patients has revealed a relative gap between the categories of certain variables, such as place of residence, drug adherence, and INH prophylaxis (Figs. 7, 8, and 9) respectively.

### Predictors of HIV infected under-five children's deaths (mortality)

In bi-variable Cox proportional regression analysis, age of the child, age of caregiver, status of parents, residence, OI at baseline, CD4 count or percent, underweight, wasting, ART adherence status, CPT prophylaxis status, INH prophylaxis status, OI’s during follow up period, Baseline Hemoglobin (Hgb) level, and Baseline WHO Clinical stages were found to have a *p*-value of < 0.25 which were candidate variables for multivariable Cox proportional hazard analysis. From the final multivariable Cox proportional regression analysis, residence, ART adherence status, INH prophylaxis, and hemoglobin (Hgb) level were found to be predictors of death at 5% of the level of significance. The hazard of death among HIV-positive under-five children who live in rural areas was 3.3 times higher (AHR 3.32:-95% CI 1.17–9.39) as compared to those who live in urban areas. The hazard of death among under-five children with poor adherence to ART medications was 3.4 fold higher than those children with good adherence (AHR = 3.36; CI: 1.06, 10.69). Furthermore, the risk of death in children under the age of five without a history of INH prophylaxis was 3.2 times higher (AHR = 3.15; CI: 1.11, 8.94) than in children with INH prophylaxis. Lastly, the hazard of death among anemic under five children was three-fold (AHR: 3.05, 95% CI: 1.16, 8.03) higher as compared to non-anemic under five children (Table 1).

**Table 1:** Bivariable and Multivariable Cox regression analysis of predictors of Death among HIV infected under five children receiving ART in West Amhara Referral Hospitals between January 2010 and December 2019

Variable	Category of Variables	Outcome status		CHR [95%CI]	AHR[95%CI]	*P*-value
		Death	Censored			
Age of the child	< 12 months	3	28	2.40(0.71, 8.08)	2.17(0.47, 10.02)	0.321
	12 -59 months	22	362	1	1	
Age of caregiver	18–30 years	9	179	1	1	
	31–40 years	9	150	1.22(0.48, 3.08)	0.58(0.18, 1.82)	0.347
	41–50 years	3	41	2.28(0.61, 8.56)	1.20(0.24, 6.05)	0.824
	> 50 years	4	20	7.88(2.35, 26.48)*	0.76(0.14, 4.19)	0.754
Status of parents	Both alive	17	304	1	1	
	mother alive but father dead	2	29	1.31(0.30, 5.67)	0.86(0.16, 4.63)	0.859
	Mother dead, but father alive	3	13	4.16(1.2, 14.25)*	1.72(0.37, 8.13)	0.491
	Both dead	3	44	2.34(0.68, 8.11)	0.75(0.16, 3.46)	0.715
Residence	Urban	11	274	1	1	
	Rural	14	116	3.85(1.73, 8.58)*	**3.32(1.17, 9.39)**	0.024**
OI at baseline	Yes	22	239	4.52(1.35, 15.12)*	1.79(0.35, 9.11)	0.483
	No	3	151	1	1	
CD4 count or percent	Above the threshold	5	179	1	1	
	Below the threshold	20	211	2.28(0.91, 5.73)*	0.96(0.32, 2.91)	0.942
ART adherence status	Good	11	308	1	1	
	Fair	6	51	3.04 (1.12, 8.22)	1.88(0.53,6.7)	0.329
	Poor	8	31	6.00(2.40, 15.01)*	**3.36(1.06, 10.69)**	0.040**
CPT	Yes	20	352	1	1	
	No	5	38	4.50(1.66, 12.19)*	1.81(0.50,6.58)	0.366
IPT	Yes	6	192	1	1	
	No	19	198	4.67(1.86, 11.75)*	**3.15(1.11, 8.94)**	0.031**
OI’s during follow up	Yes	19	159	2.79(1.11, 7.049)*	1.51(0.52, 4.34)*	0.446
	No	6	231	1	1	
Baseline hemoglobin(Hgb)	Anemic(< 10 g/dl)	12	39	7.40(3.37, 16.23)*	**3.05 (1.16, 8.03)**	0.024**
	Nonanemic(≥ 10 g/d)	13	351	1	1	
Baseline WHO Clinical Stages	Mild (stage I & II)	5	218	1	1	
	Advanced (stage III&IV)	20	172	5.00(1.88, 13.36)*	1.15(0.27, 4.86)	0.845

## Discussion

This institution-based retrospective follow-up study was conducted in West Amhara Referral Hospitals, Northwest Ethiopia to assess the survival time and predictors of death. In this study, the overall incidence rate of death was 2.87 deaths per 1000 child-months (95% CI: 1.94–4.25) during the follow-up period. This finding is lower when compared with a study conducted in Oromia Liyu Zone, Amhara Region, Ethiopia (IR: 5.9 deaths per 100 child-months) [25] and in the Karonga district of northern Malawi, in which HIV infected under five Child incidence rate was 16.6/1000 PYO [11]. The variations might be due to the differences in clinical characteristics of the study participants and study settings, as the current study included only referral hospitals. The above disparity could possibly be attributable to differences in ART enrollment periods, as a prior study in northern Malawi's Karonga district looked at children who started ART earlier (before 2012) and had different treatment eligibility criteria. Since 2013, the WHO has made significant adjustments to the recommended pediatric age for ART initiation, regardless of clinical or immunologic state, based on data indicating early ART initiation reduces HIV-related morbidity and mortality in children [26].

In this study, a high number of deaths10,(40%) occurred within the first one year after starting ART. This finding is similar to the studies conducted in Oromia Liyu Zone, Amhara Region, Ethiopia [25] Gonder [27], Ngeria [28], Cameroon [29] and Sub-Saharan Africa [12], reporting that the death rate was high in early times of ART initiation. This could be due to undiagnosed immune reconstitution inflammatory syndrome (IRIS),a common side effect of ART, especially in patients with advanced disease stages and low CD4 cell counts. According to a study conducted in South Africa, IRIS affected 21% of children starting ART, and IRIS was responsible for one-quarter of the mortality in the first six months [30]. Additionally, based on a study conducted in Japan, 50% of ART-associated IRIS occurred within the first month of ART initiation and this might cause higher early mortality [31].

Higher premature mortality in this study also might be associated with INH prophylaxis of included participants, as more than half (52.7%) of the study participants had not received IPT. The reason for not taking IPT might be due to the presence of OIs (attacked by different types of OIs), which are the most common causes of premature death.

The cumulative probability of survival of children on ART in this study was 85% at the end of the follow-up period (95%CI; 76.42–91.25). This was comparable with the report of a study conducted in Oromia Liyu Zone, Amhara Region, Ethiopia, in which the cumulative probability of survival was 87% [25]. The possible elucidation may be due to almost similar follow-up periods (follow-up times) for children after starting ART.

In this study, residence of children is one of the predictors of reducing the survival of children on ART. Living in a rural area increases the risk of death by 3.3 times more than living in an urban area. This is similar to the study conducted in Wolaita zone health facilities in Ethiopia and Tanzania [32, 33]. This similarity shows that mortality rates in urban areas are consistently lower than in rural areas in child mortality. This could be due to poor hygiene and sanitation; malnutrition is prevalent in rural areas; poor knowledge of how to care for HIV and others [34].

Additionally, the study found that ART drug adherence was another important predictor of mortality. Individuals who had poor ART adherence levels were more at risk of dying than individuals who had good adherence levels. Adherence status of the child to medications throughout the follow-up time is an important predictor in agreement with the majority of studies. This finding is in line with a study conducted at Oromia Liyu Zone, Amhara Region, Ethiopia; Gamo Gofa Zone, Southern Ethiopia; and Wolaita zone health facilities [4, 25, 32]. The possible elucidation may be because the relationship between adherence and mortality is directly forward. Those factors which may contribute to poor adherence will indirectly cause the death of patients from their regular follow-up. Furthermore, poor adherence to ART leads to virologic, immunologic, and clinical failure that is mediated mainly by two potentially reinforcing mechanisms. Poor adherence to ART leads to failure to suppress viral replication, thus increasing the likelihood of developing HIV mutations that could lead to the development of drug-resistant viral strains. Also, poor adherence to ART fails to prevent further viral destruction of the cellular immune system, with a consequent reduction in the level of CD4 + cells and the development of opportunistic infections. Based on the findings of a qualitative study conducted in Uganda, the principal factors for poor adherence were poverty, presence of drug side effects, depression, poor peer support and counseling, stigma and discrimination, which indirectly contribute to the increased incidence of treatment failure and death [35].

The INH prophylaxis status of children under the age of five was one of the significant determinants of death. Children with no history of INH prophylaxis were associated with reduced survival. This finding was consistent with findings in a study done in the Amhara Region, Ethiopia [25], and Gamo-Gofa (Ethiopia) [4]. The possible reason for such a higher level of death in these under-five children without INH prophylaxis during ART enrolment could be the occurrence of some OIs like pneumocystis pneumonia, toxoplasmosis, tuberculosis, and recurrent diarrheal diseases. If there were OIs present for the child, INH prophylaxis could not be given.

This study also found that the risk of mortality among anemic HIV-positive under-five children was three-fold higher as compared to their non-anemic counterparts. Previous Ethiopian studies also documented that anemia had a significant impact on the survival of HIV-infected children [13, 27, 32]. Additionally, studies from other sub-Saharan African countries also demonstrated that low hemoglobin was a predictor of mortality [15, 33, 36]. The most common cause of anemia among people living with HIV is the side effects of zidovudine, of which 53.5% of this study's participants were taking a combination of 4c (AZT-3TC-EFV) or 4d (AZT-3TC-NVP) during the follow-up time. One of the most common causes of anemia (megaloblastic anemia) among HIV-infected patients is AZT [37, 38].

### Limitations of the study

This study did not incorporate important predictors like viral load, income, immunization status of the children, micronutrient deficiency due to an incomplete recording system, and provider and system-related factors with mortality. Furthermore, early mortality might be under estimated since the study excluded children who had ART follow-up of less than 3 months.

## Conclusion and recommendations

The incidence rate of death is 2.87 deaths per 1000 child-months and the cumulative incidence of death is 6.02%. The cumulative probability of survival of under-five children on ART after the last month of follow-up was 85%. The death rate of HIV-infected under-five children on ART is high within the first one year after enrolment. The risk of death increased if the child's residence was in a rural area, had poor ART adherence, lacked Isoniazide prophylaxis, and was present with anemia.

Based on the findings of this study, the following recommendations were made.

### For healthcare providers

o Health care providers shall give special emphasis and close follow-up for HIV positive under-five children especially, within the first year of enrolment.
p To address barriers to poor adherence, healthcare providers must strengthen their collaborative work with adherence counsels, and patients with poor adherence must have frequent follow-up schedules.
q All eligible HIV-positive patients should be given IPT if there are no contraindications because it has numerous advantages which indirectly improve patient survival.

### To Referral Hospitals in West Amhara (UoGCSH, FHCSRH, DTCRH, and DMCRH)

o Additional follow-up visits and close monitoring will be facilitated and made available, in the first year after ART initiation, the highest incidence of death occurs during this time.
p Special consideration shall be given to improving the adherence levels of ART by improving adherence counseling strategies.

### Amhara Public Health Institute (APHI), Regional Health Bureau (RHB), and the Federal Ministry of Health (FMOH)

o All of the aforementioned stakeholders must increase close monitoring and supportive supervision of ART sites in such areas.

## Data Availability

The data set used and analyzed during the current study is available from the corresponding author on reasonable request.

## Abbreviations

AHR: Adjusted Hazard Ratio
AIDS: Acquired Immune Deficiency Syndrome
ART: Antiretroviral Therapy
ARV: Anti-Retro Viral
AZT: Zidovudine
CD4: Cluster of Differentiation 4
CI: Confidence Interval
CPT: Cotrimoxazole Preventive Therapy
HAART: Highly Active Antiretroviral Therapy
HIV: Human Immunodeficiency Virus
IPT: Isoniazid Preventive Therapy
LMICs: Low and Middle Income Countries
MTCT: Mother to Child Transmission
NVP: Nevirapine
OIs: Opportunistic Infections
PLHIV: Patient living with Human Immunodeficiency Virus
PYO: Person Years Observation
SSA: Sub-Saharan Africa
TB: Tuberculosis
WHO: The World Health Organization
